# Contemporary Theory and Research

**Published:** 1893-04-01

**Authors:** 


### Contemporary Theory and Research.

### WHY ARE PARISIANS UNDER-SIZED?

M. Delthil urges upon Parisians the necessity for a regular supply of milk from the country, on the ground that the alimentation of stabled cows, though it often results in a more abundant supply of milk, is greatly to the detriment of its mineralisation. He attributes the continued decadence of Parisians as regards stature to the bad system of infant feeding in vogue, and specially to the deficiency of the milk diet. Parisian mothers, it seems, rarely suckle their own children, being either too grand or, in humble ranks of life, too busy. He considers infants are thrown upon solid diet too early, or nourished with poor Parisian milk deficient in food value. He concludes: "In my numerous observations of the antecedents of children afflicted with affections of the osseous tissue, white swellings, &c., I have noticed that in the immense majority of these cases these patients had been victims of bad nourishment at an early age—either bad nurses or insufficient milk food, or milk diet discontinued too soon—whence insufficiency of phosphates for the development of the osseous tissue." M. Delthil even traces the low stature of races living in barren regions to the poverty of the milk supply.

### THE DANGERS OF BATHING.

Herr Jaeger furnishes in the "Zeits-chrift für Hygiene" a report of his researches into the etiology of the infectious fever known as the Weil's-che Krankheit, which has been puzzling the German doctors. The source of the mischief seems to be in many cases bathing in polluted water. Investigating many cases of typhoid which arose in Ulm among the soldiers, Herr Jaeger found that the military bathing-place was situated just below the point where the Danube is joined by the highly-polluted river Blau. This stream is described as practically an open sewer, and even before it reaches Ulm is grossly contaminated in its flow through the small village of Söflingen. He further ascertained that in this village a mysterious disease had been rife among ducks and geese, while fowls were also occasionally attacked, and that, moreover, it was a common custom to throw the dead carcases of these animals into the Blau as the readiest means of getting rid of them. A careful examination of the birds which had succumbed to this disease revealed the constant presence of a micro-organism, which Jaeger asserts to be identical with that found by him repeatedly, and isolated in the cases of typhoid investigated at Ulm. Mixing some of the polluted Blau water at Söflingen with sterile broth and inoculating white mice with it, they were killed in sixteen minutes, and organisms were found abundantly present in various parts of their bodies, which were identical with those found in the birds and in the Ulm cases. It is not necessary to suppose that the germ was communicated through the pores of the skin while bathing, since few persons swim or dive without swallowing a certain amount of water, and even a few drops would be sufficient.

### THE RECURRENCE OF ERUPTIVE DISEASES.

M. Ramonat addresses a note to the Société de Medicine on the interesting subject of the recurrence of eruptive complaints. He admits the frequent recurrence of measles, but considers it to be very rare in scarlatina. He says: "I have seen numerous cases of very pronounced recurrence of measles, and only doubtful cases of scarlatina. In general the second attack of the measles is less pronounced than the first. Like most observers who have noted these cases, I have found that a recurrence of measles takes place very rapidly, after an interval of one or two years only.

### EPIDEMICS IN AFRICA.

Dr. Schoop, a ship doctor, has been making observations on the epidemic diseases most frequent among the negroes of the West Coast of Africa. Small-pox develops frequently with great intensity in the black race; the negro is but slightly affected by vaccination, of which, moreover, he has a great horror. In the Canary Isles especially small-pox is very prevalent. The West Coast of Africa, from the Canaries to the mouth of the Congo, has been very often the seat of yellow fever, but for the last ten years there has been no epidemic, owing, Dr. Schoop thinks, to the improvement in sanitary matters introduced by European colonists. The plague is rare in Africa, and it was noted that any outbreak of typhoid fever coincided generally with the arrival of new troops from France. Dr. Schoop considers that endemic maladies, demanding for their development a certain length of residence on the coast, need not be dreaded by expeditions remaining only a limited time at each station.

### CHLORALOSE.

A new hypnotic, offering neither inconvenience nor danger, has just made its appearance in the "Comptes Rendus" of the Académie de Sciences, under the auspices of M. Hanriot and M. Richet. In the research for substances capable of slowly introducing chloral into the organism they were first led to study chloralides, and specially lactic chloralide. But lactic chloralide proved to have no hypnotic qualities, and to be productive of serious symptoms ending in asphyxia. They obtained, on the contrary, excellent results from a body resulting from the combination of chloral with glucose, "anhydrogluco-chloral," which they propose to call chloralose. The physiological properties of chloralose are very interesting, for it is a substance possessing two effects, which are apparently contradictory; it is hypnotic, and it increases the excitability of the spinal cord. Its therapeutic qualities are said to have been well established by frequent administration in cases of obstinate insomnia, when it has often succeeded where other remedies have been tried in vain.

